# Supplementary figures and images for: Protist Diversity and Metabolic Strategy in Freshwater Lakes Are Shaped by Trophic State and Watershed Land Use on a Continental Scale
Source: mSystems. 2022 Jun 22;7(4):e00316-22. doi: 10.1128/msystems.00316-22 (PMC9426515; doi:10.1128/msystems.00316-22)

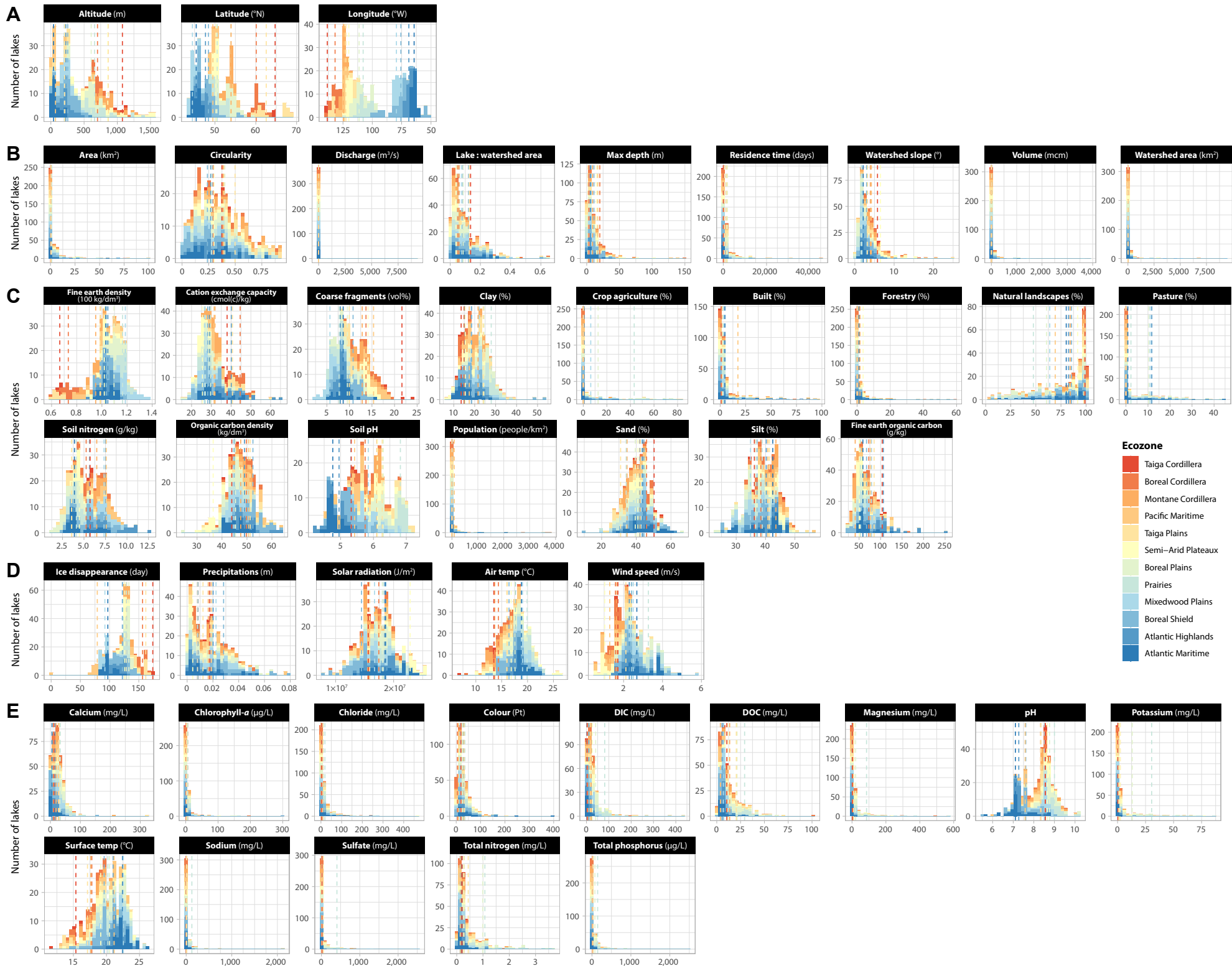

Supplement: FIG S1 [file msystems.00316-22-s0001.pdf]

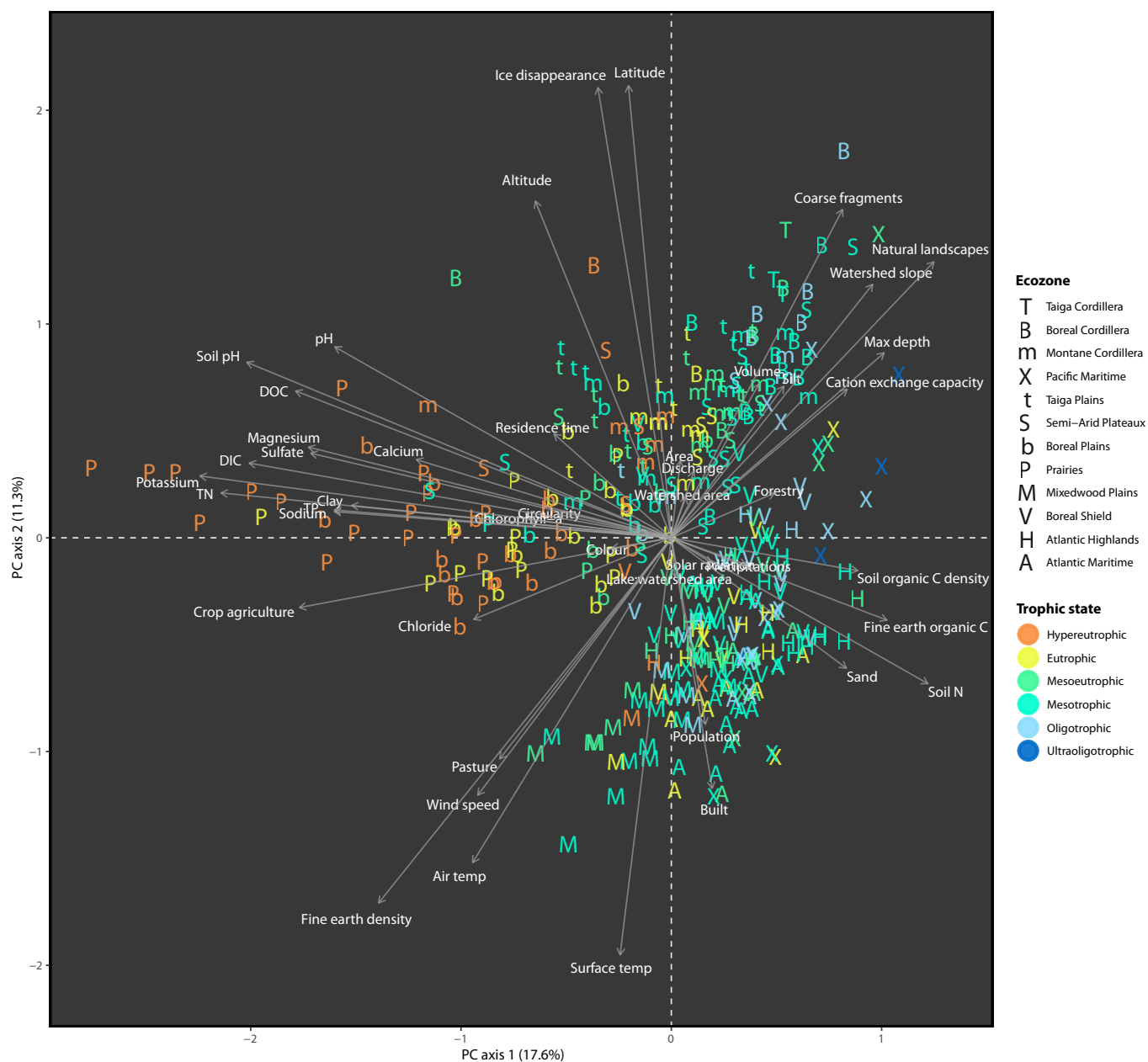

Supplement: FIG S2 [file msystems.00316-22-s0002.pdf]

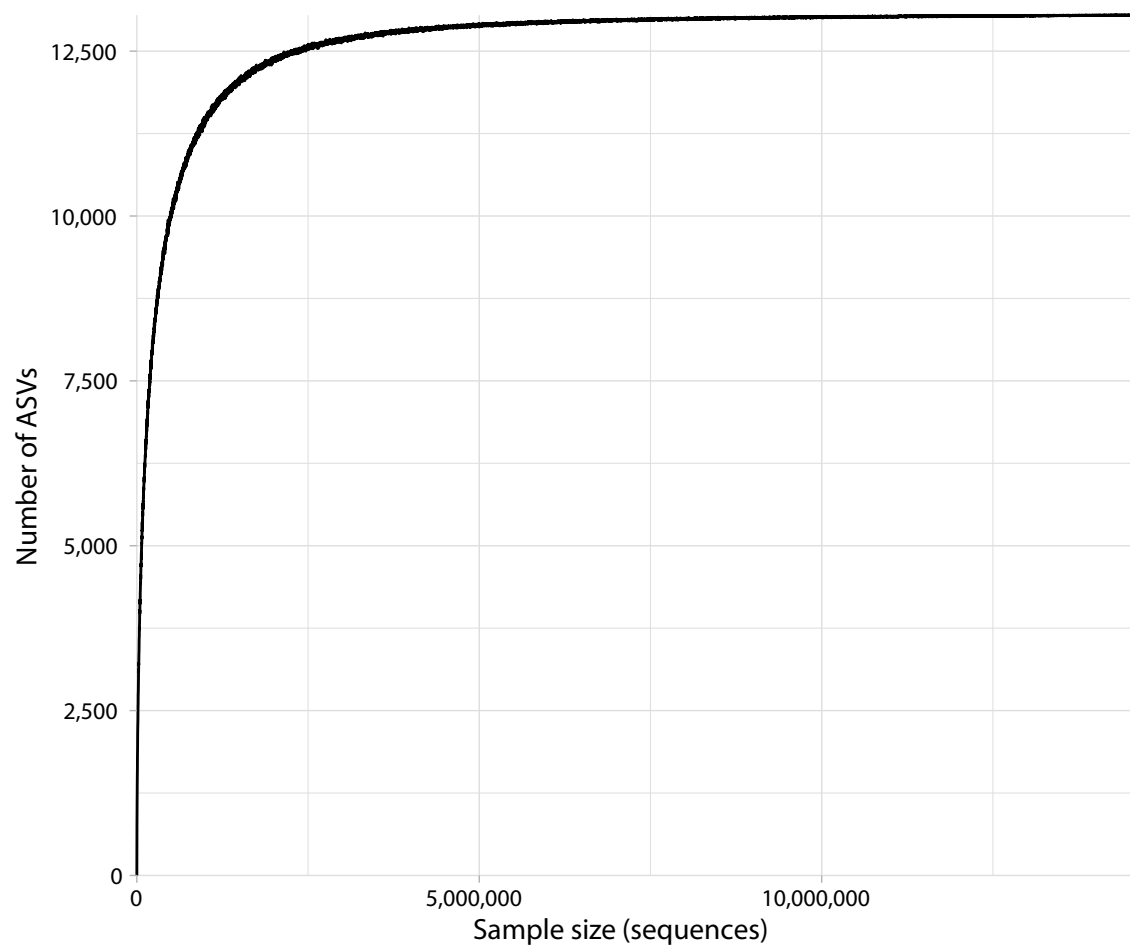

Supplement: FIG S3 [file msystems.00316-22-s0003.pdf]

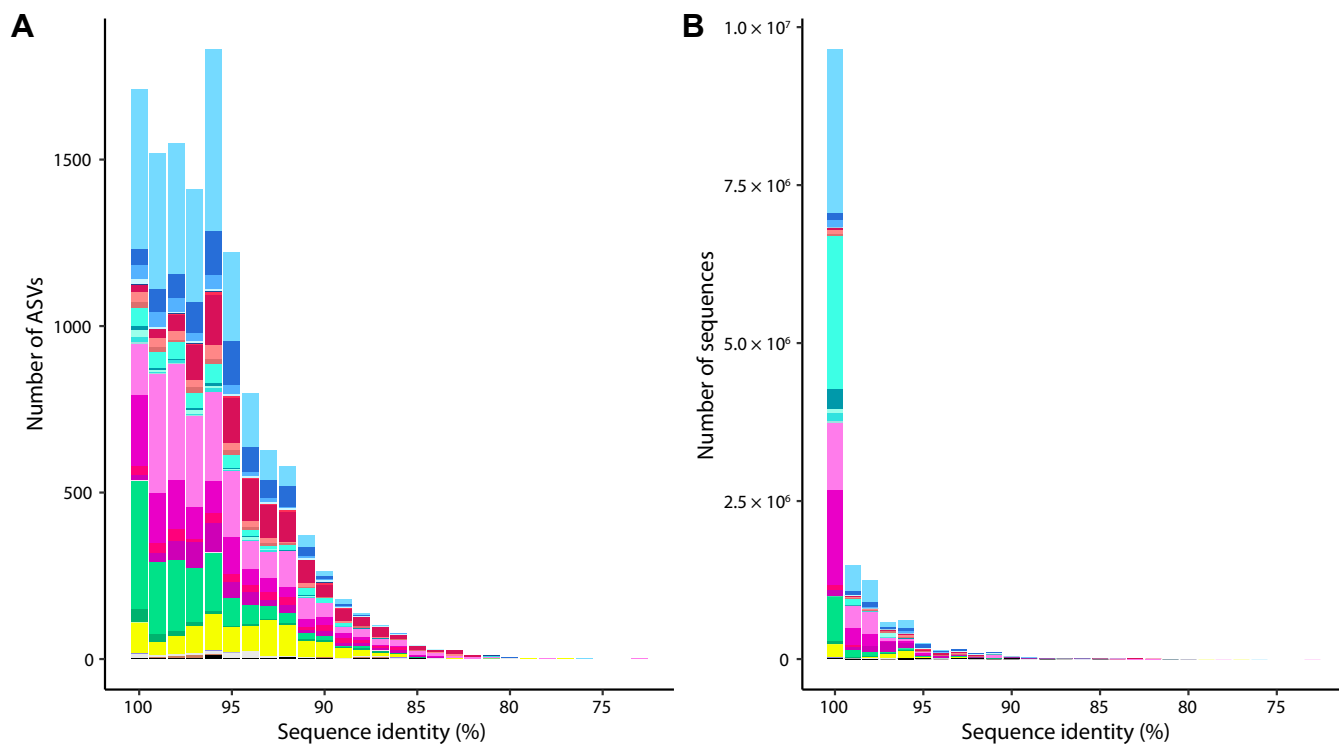

**PR<sup>2</sup> taxonomy**

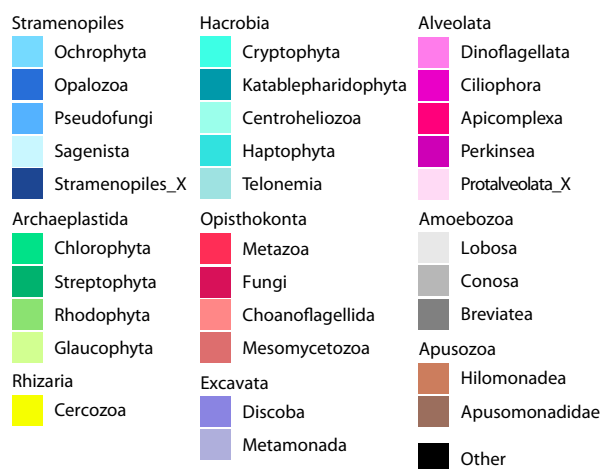

Supplement: FIG S4 [file msystems.00316-22-s0004.pdf]

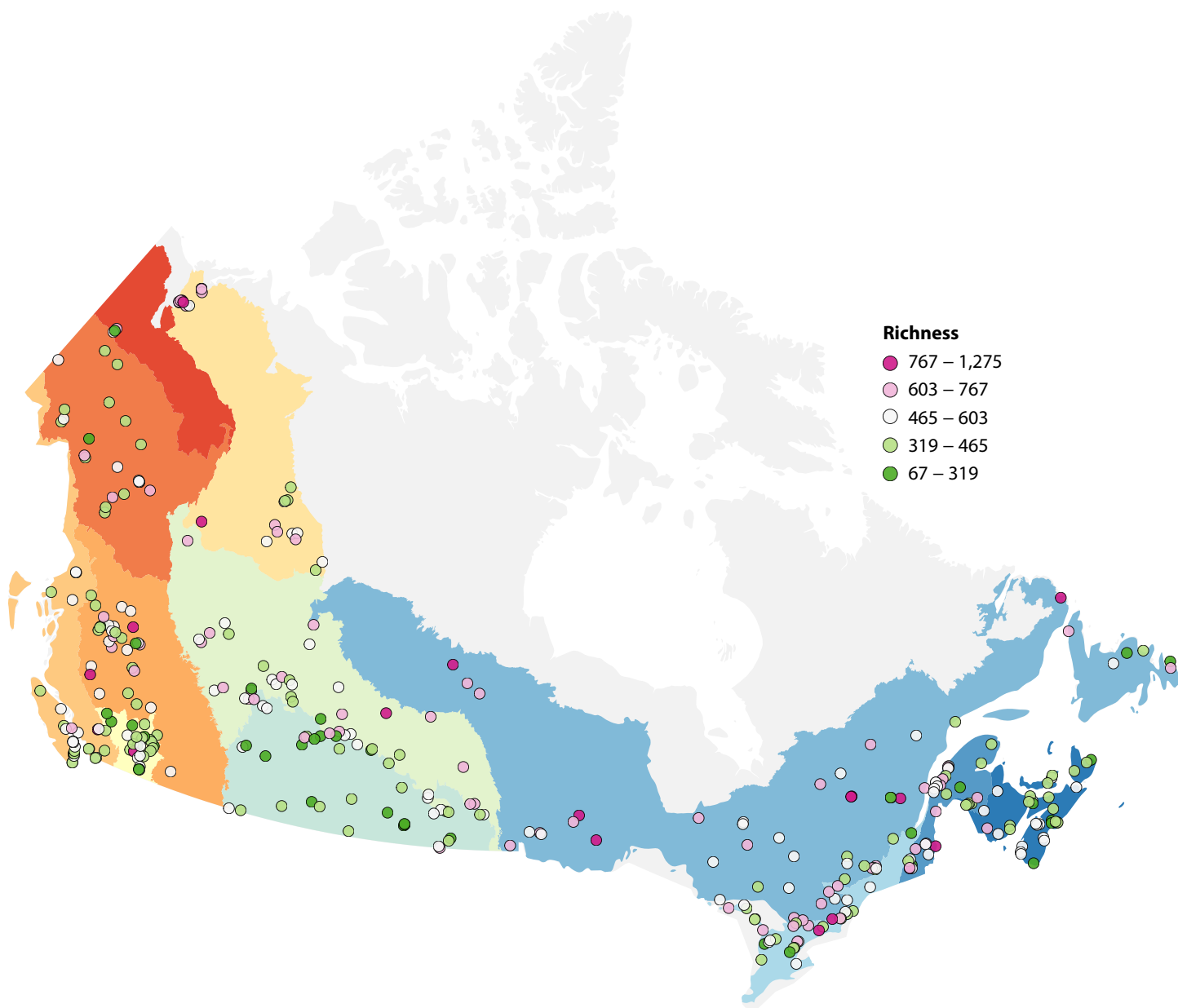

Supplement: FIG S5 [file msystems.00316-22-s0005.pdf]

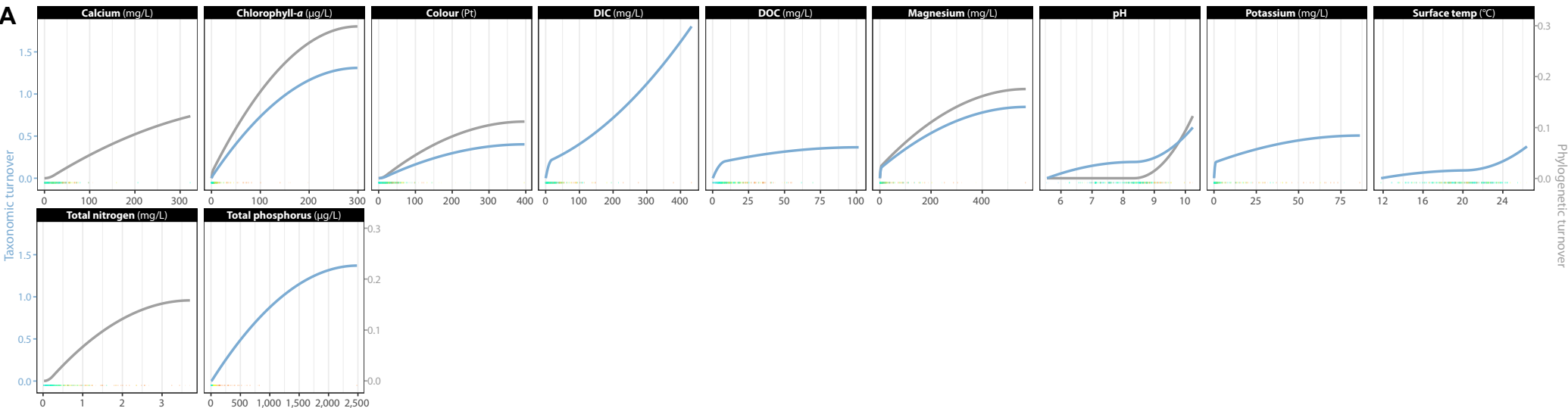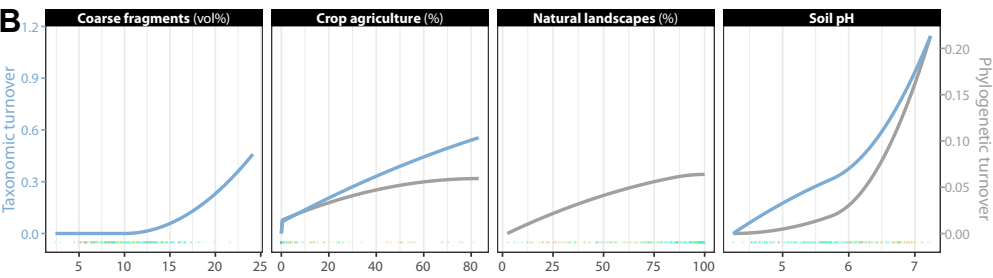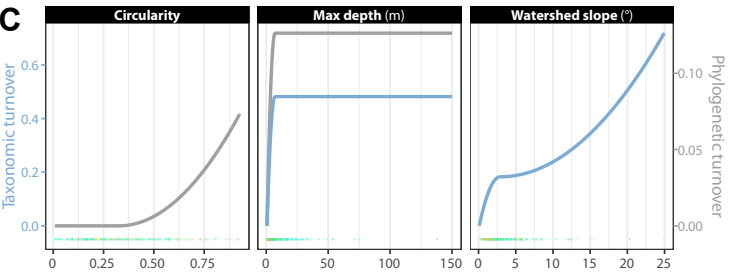

Supplement: FIG S6 [file msystems.00316-22-s0006.pdf]

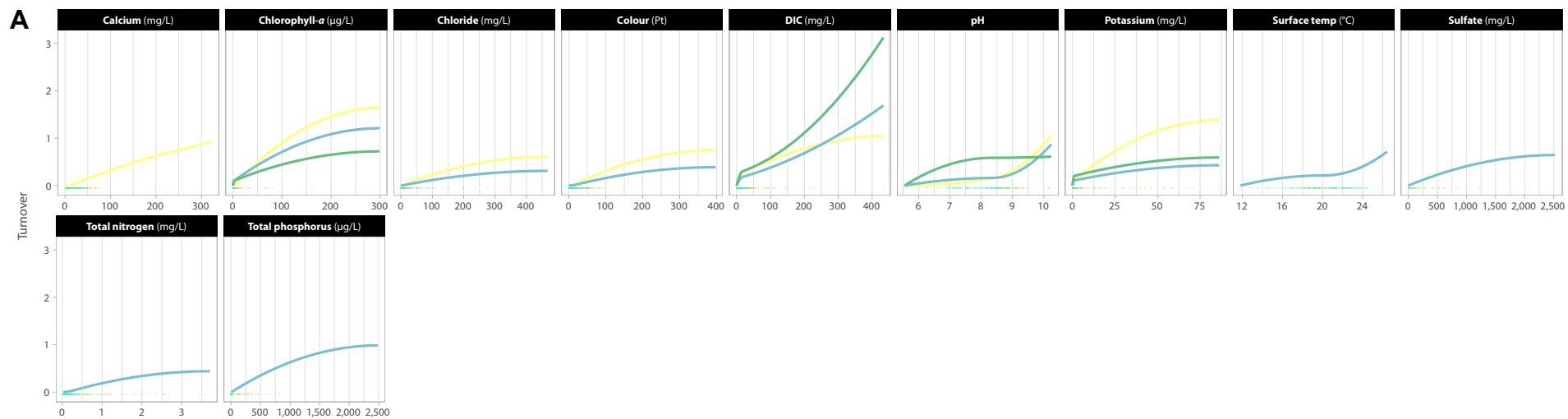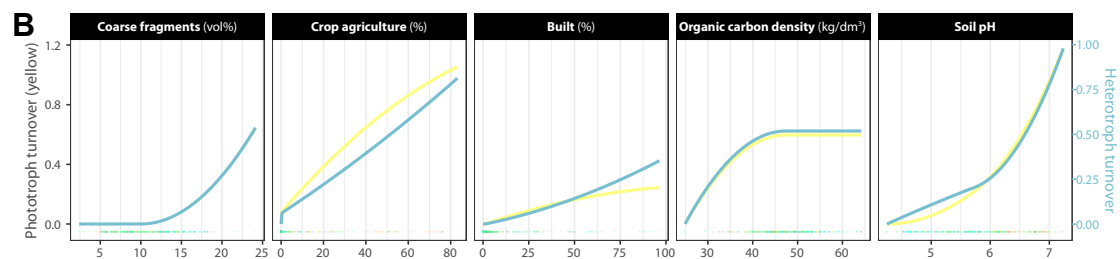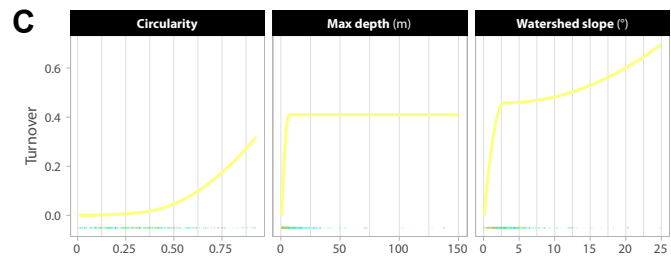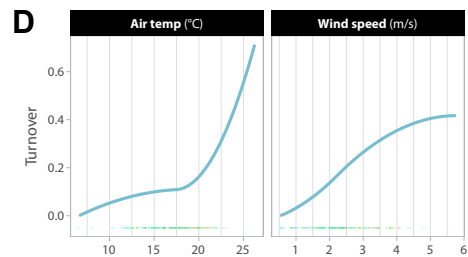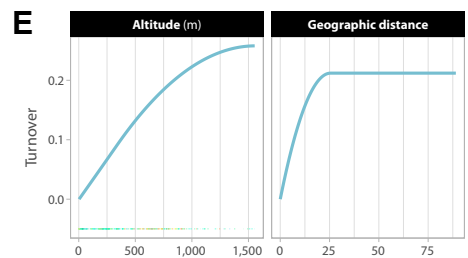

**Turnover**

Phototrophs  
Heterotrophs  
Mixotrophs

Supplement: FIG S8 [file msystems.00316-22-s0008.pdf]
