# Supplementary material for: Protist Diversity and Metabolic Strategy in Freshwater Lakes Are Shaped by Trophic State and Watershed Land Use on a Continental Scale
Source: mSystems. 2022 Jun 22;7(4):e00316-22. doi: 10.1128/msystems.00316-22 (PMC9426515; doi:10.1128/msystems.00316-22)
Supplement: TABLE S2 [file msystems.00316-22-s0010.docx]

| Response variables | Explanatory variable category | Deviance explained (%) | Predictors |
| --- | --- | --- | --- |
| All protists (taxonomy) | Physicochemistry | 38.4 | DIC (1.82), TP (1.37), chlorophyll-*a* (1.31), magnesium (0.85), pH (0.61), potassium (0.51), colour (0.4), surface temperature (0.38), DOC (0.37) |
|  | Watershed | 15.4 | Soil pH (1.16), crop agriculture (0.56), coarse fragments (0.47) |
|  | Morphometry | 6 | Watershed slope (0.72), maximum depth (0.48) |
|  | Weather | NS | – |
|  | Geography | NS | – |
| All protists (phylogeny) | Physicochemistry | 32.9 | Chlorophyll-*a* (0.3), magnesium (0.18), TN (0.16), pH (0.13), calcium (0.12), colour (0.11) |
|  | Watershed | 15.7 | Soil pH (0.22), natural landscapes (0.06), crop agriculture (0.06) |
|  | Morphometry | 8.4 | Maximum depth (0.13), circularity (0.07) |
|  | Weather | NS | – |
|  | Geography | NS | – |
| Phototrophs* | Physicochemistry | 34.9 | Chlorophyll-*a* (1.64), potassium (1.38), pH (1.08), DIC (1.05), calcium (0.92), colour (0.75), chloride (0.61) |
|  | Watershed | 20.1 | Soil pH (1.19), crop agriculture (1.06), soil organic carbon density (0.6), built (0.24) |
|  | Morphometry | 5.2 | Watershed slope (0.7), maximum depth (0.41), circularity (0.32) |
|  | Weather | NS | – |
|  | Geography | NS | – |
| Heterotrophs* | Physicochemistry | 42.1 | DIC (1.7), chlorophyll-*a* (1.21), TP (0.99), pH (0.89), surface temperature (0.73), sulfate (0.64), TN (0.44), potassium (0.43), colour (0.39), chloride (0.31) |
|  | Watershed | 18.9 | Soil pH (0.99), crop agriculture (0.82), coarse fragments (0.55), soil organic carbon density (0.52), built (0.36) |
|  | Morphometry | NS | – |
|  | Weather | 3.2 | Air temperature (0.73), wind speed (0.42) |
|  | Geography | 2.2 | Altitude (0.26), geographic distance (0.21) |
| Mixotrophs* | Physicochemistry | 18.5 | DIC (3.15), chlorophyll-*a* (0.72), pH (0.61), potassium (0.59) |
|  | Watershed | NS | – |
|  | Morphometry | NS | – |
|  | Weather | NS | – |
|  | Geography | NS | – |
