# Supplementary material for: Protist Diversity and Metabolic Strategy in Freshwater Lakes Are Shaped by Trophic State and Watershed Land Use on a Continental Scale
Source: mSystems. 2022 Jun 22;7(4):e00316-22. doi: 10.1128/msystems.00316-22 (PMC9426515; doi:10.1128/msystems.00316-22)
Supplement: FIG S7 [file msystems.00316-22-s0007.pdf]

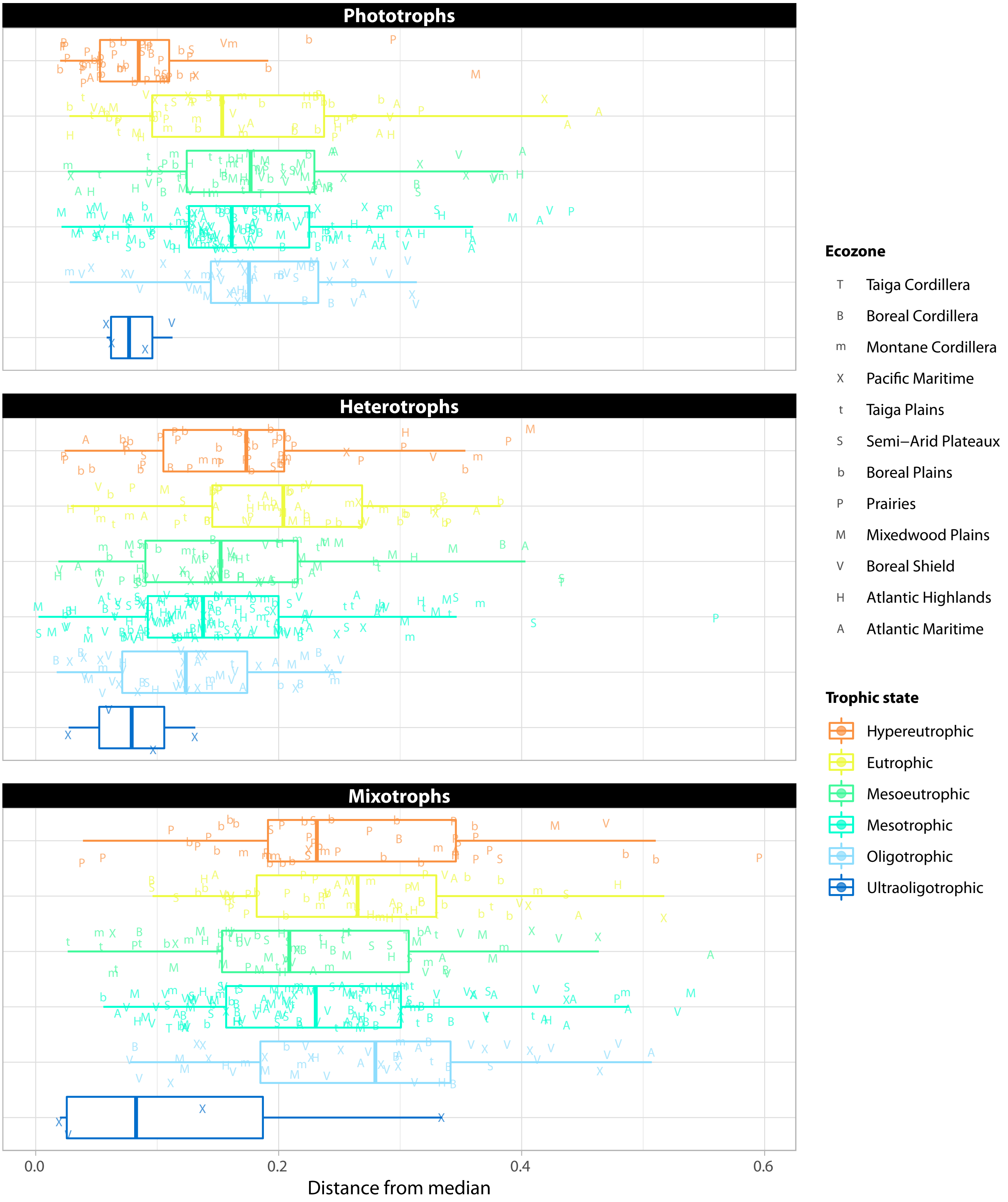

Phototrophs
Heterotrophs
Mixotrophs
0.0
0.2
0.4
0.6
Distance from median
Ecozone
T Taiga Cordillera
B Boreal Cordillera
m Montane Cordillera
X Pacific Maritime
t Taiga Plains
S Semi–Arid Plateaux
b Boreal Plains
P Prairies
M Mixedwood Plains
V Boreal Shield
H Atlantic Highlands
A Atlantic Maritime
Trophic state
Hypereutrophic
Eutrophic
Mesoeutrophic
Mesotrophic
Oligotrophic
Ultraoligotrophic
